# Perfectionism, mattering and loneliness in young adulthood of Generation-Z

**DOI:** 10.1016/j.heliyon.2023.e23330

**Published:** 2023-12-05

**Authors:** Bushra Shafiq, Anam Ali, Hidna Iqbal

**Affiliations:** a Department of Developmental & Behavioral Pediatrics, University of Child Health Sciences- The Children's Hospital (UCHS-CH), Lahore, Pakistan; b Centre for Clinical Psychology, University of the Punjab, Lahore, Pakistan

**Keywords:** Generation-Z, Perfectionism, Mattering, Loneliness, Young adults

## Abstract

**Introduction:**

Young adulthood is a transitional period where adults are faced with new social and psychological roles. So, in order to matter and to form relationship with others, they become vulnerable to set exaggerated high standards for themselves and others, which can eventually lead them to experience loneliness.

**Objectives:**

The present study aimed to determine the relationship of perfectionism, mattering with loneliness in young adults of Gen-Z after controlling for covariates. It was also aimed to find out gender differences on perfectionism, mattering and loneliness in young adults.

**Method:**

Correlational research design was used. A sample of 320 students with age ranging from 18 to 24 years, was collected from seven different government and private universities of Lahore through purposive sampling. The sample comprised of 160 male students and 160 female students. Multidimensional perfectionism scale, Mattering scale and UCLA loneliness scale-3 were administered to measure perfectionism, mattering and loneliness respectively.

**Results:**

Pearson product moment correlation showed that closer relationship of young adults with father (p < .01), mother (p < .01) and friends (p < .05) had a significant negative relationship with loneliness. Results of hierarchical regression analysis showed that age was significant negative predictor of loneliness. Self-oriented, other-oriented and socially prescribed perfectionism were significant positive predictors of loneliness. Mattering was significant negative predictor of loneliness. Independent sample *t*-test showed no significant differences between male and female students on perfectionism, mattering and loneliness.

**Conclusion:**

Perfectionist individuals tend to set stringent standards for themselves and others as well. The more they engage in perfectionism, the more they tend to feel that they do not matter to others. Feeling of not mattering to others can possibly lead the person to experience loneliness.

## 1 Introduction

Generation-Z (Gen-Z) refers to any individual born from 1997 onwards with the eldest Gen-Z being 25 years old to-date. In urban population, most of the Gen-Z members are currently enrolled in college or university. Moreover, they have the highest likelihood of being in college which is considered as period of young adulthood [1]. Young adulthood is a transitional period comprising of youthful searching, change, inspection, and exploration. Young adults are faced with new social and psychological roles regarding their independence, education, employment, making new relationships and sometimes even moving into parenthood [2]. Usually, there are societal and parental pressures on them regarding these responsibilities. Due to the expectations of parents, persuasion of society, young adults often set elevated standards and unattainable goals for themselves in order to be best and gain approval from others which is considered as perfectionism [3].

Perfectionism is an adherence to exaggerated and excessive high standards and intense need to perform flawlessly [4]. It is striving for excellence taken to the paramount level. Individual with perfectionist tendency tend to put pressure on themselves believing that they should be able to attain impossible [5].

Hewitt and Flett proposed a multidimensional model of perfectionism consisting of self-oriented, other-oriented and socially prescribed perfectionism. Self-oriented perfectionism involves having elevated standards for oneself and appraising one's performance rigorously [6]. It also involves a motivational aspect which is demonstrated by individual's striving for keeping away from failure and also for achieving perfection in one's aspirations [7]. Other-oriented perfectionism refers to demanding and requiring from others to be perfect and ideal and assessing their behaviors critically. It is the dimension of perfectionism which is directed outward [8]. Socially prescribed perfectionism can be explained as the belief of the person, that other demand from them to be perfect and have ideal goals for them. Moreover, such individuals also exhibit the need to gain attention and approval of others. Thus, perfectionism is a multidimensional personality trait with both intrapersonal and interpersonal components [7]. People with perfectionism tend to set stringent standards for themselves and others as well. They seek flawlessness which reflects the need to seek approval of others and the need to matter to others [9].

Elliot et al. described mattering as “the perception that, to some degree and in any of variety ways, we are a significant part of the world around us.” They have defined mattering in terms of awareness (i.e., others pay attention to them), importance (i.e., others invest in them), and reliance (i.e., others depend on us for fulfilling their needs). Feelings of not mattering to others provoke a sense of unimportance and unrelatedness in the unsympathetic world which can further lead the individual to get involve in maladaptive behaviors for gaining a sense of importance and significance [10].

Mattering has been shown to lessen the psychological problems and contributes to well-being particularly in adults. Similarly, mattering, sense of being significant and important to others diminishes the problem related to maladjustment, such as depression in young adults [11]. Flett, Flett and Wekerle also discovered that negative appraisal of mattering promotes social disconnection and isolation from others [12]. As, the perception of being insignificant to others can create hindrances in making connections and forming quality relationships and can make adults to feel lonely [13]. Loneliness is a common phenomenon experienced by nearly every individual at some point in their lives. Weiss defined loneliness as a lack of intimate or quality relationships in one's life [14]. There are several risk factors for increased loneliness in adults such as lack of social connection, lack of social support from family and friends, poor attachment to parents were related to greater feelings of loneliness [13]. Previous literature suggested that individuals who generally experiences low feelings of belongingness or had poor relationship with parents or peers were more likely to experience loneliness [15]. 10.13039/100014337Furthermore, a study reported that low feelings of mattering to others was associated with loneliness in males and females [16].

A number of studies have been conducted on perfectionism and mattering [11,17]. Mattering and loneliness [13,15,16]. Research studies suggested that socially prescribed perfectionism was significantly negatively associated with mattering [17,18] and is also related to negative consequences such as anxiety and depression [19]. But as per researcher's knowledge, no research has been conducted who studied the association of perfectionism, mattering and loneliness in young adults of Gen-Z in Pakistan. Therefore, the present study examined the relationship between perfectionism, mattering and loneliness in young adults of Gen-Z. The study also provided information of clinical significance by yielding insight regarding how not mattering to others can augment feelings of loneliness which can further lead the troubled perfectionist to engage in destructive behaviors. It will also help the clinicians and therapist to understand the distressed perfectionist and to make management and intervention plan in a better way while dealing with them.

## 2 Objectives

• to determine the association of perfectionism, mattering and loneliness in young adults of Gen-Z.
• to determine the relationship perfectionism, mattering and loneliness in young adults.
• to find out gender differences on perfectionism, mattering and loneliness in young adults

### 2.1 Hypotheses

• There is likely to be association of perfectionism, mattering and loneliness in young adults of Gen-Z.
• Perfectionism and mattering are likely to be predict loneliness in young adults after controlling for covariates (age, closer relationship with father, mother and friends)
• There is likely to be gender difference on perfectionism, mattering and loneliness in young adults

## 3 Method

### 3.1 Research design

Cross-sectional research design was used in the study to investigate the relationship between perfectionism, mattering and loneliness in young adults.

### 3.2 Sample characteristics

**Fig. 1:**
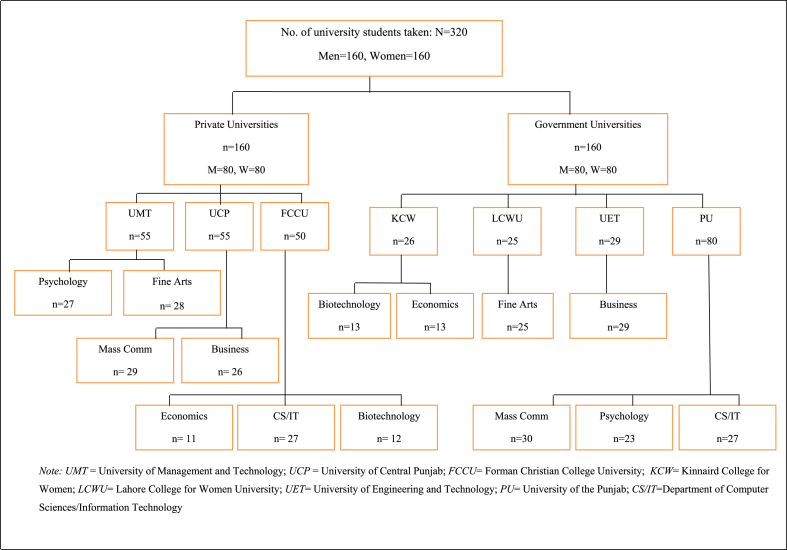
Frequency distribution of sample from different government and private universities.

Most of the Gen-Z members, with age ranging from 18 to 24 years, were currently university or college students and were born from 1997 onwards with the eldest Gen-Z being 24 years old at the time of data collection. A sample of 320 students (comprised of 160 male students and 160 female students) was collected from seven different government and private universities of Lahore, Pakistan through purposive sampling (see Fig. 1). Sample was calculated through G-power analysis using medium effect size and alpha 0.05. Mean age of male students was 21.14 years (SD = 1.63) and female students was 20.39 years (SD = 1.40). On average, male students spent 5 h studying per day whereas females spent 6 h per day. The average monthly income of the family was reported to be Rs.120928.125.

Students who are unmarried and enrolled in BS (Honors) and MS program in universities were included in the study. Students with any psychological disorder or physical disability were excluded.

## 4 Measures

### 4.1 Multidimensional perfectionism scale (MPS)

A 45-items scale of MPS was used to measure the multidimensional perfectionism including self-oriented, other-oriented, and socially prescribed perfectionism. There were 15 items for measuring each dimension. Items 8 (I never aim for perfection on my work) is an item of self-oriented perfectionism. Each item was rated on 7-points Likert scale. The scale ranges from strongly disagree to strongly agree. Higher the score on the scale, higher will be levels of perfectionism. Cronbach alpha for the subscales; self-oriented perfectionism, socially prescribed perfectionism, other oriented perfectionism was .86, .87, and 0.82 respectively [6]. The reliabilities for the three subscales; self-oriented perfectionism, socially prescribed perfectionism, other oriented perfectionism for the present study were 0.87, 0.83, and 0.81 respectively and for multidimensional perfectionism scale it was 0.90.

### 4.2 The mattering scale

Mattering scale was used to measure the extent to which one feels that they matter and the perception of being significant to others. It has three components including awareness, importance, and reliance. Item 9 (people are usually aware of my presence) is an item related to awareness. The response on each item was rated across 7-points Likert scale with responses marked from (1) strongly agree to (7) strongly disagree. The alpha coefficient for the subscales; awareness, importance and reliance were 0.82–87, 0.79 to 0.85, and 0.83 to 0.87 respectively and for the mattering scale alpha coefficient ranges from 0.88 to 0.92 [10]. The reliabilities for the three subscales; awareness, importance and reliance for the present study were 0.86, 0.88, and 0.74 respectively and for the mattering scale it was 0.91.

### 4.3 UCLA Loneliness Scale Version-3

It was a 20 items questionnaire, used to measure the subjective feelings of loneliness and social isolation. Item 4 is “how often do you feel alone?” The responses were rated on 4 points Likert scale ranging from 1 (often) to 4 (never). The high scores on the test indicated high feelings of loneliness among individuals [20]. UCLA has alpha coefficient ranging from 0.89 to 0.94. The reliability for the UCLA Loneliness Scale Version-3 in the present study was found to be 0.92.

### 4.4 Demographic questionnaire

A demographic questionnaire was included to gather information regarding the participant's age, birth order, gender, number of siblings, education, institution and department, religion, monthly income, family system, nature of relationship with friends and parents and how often do they go to parties and hang-out with friends, etc.

### 4.5 Procedure

After approval from institutional review board of University of the Punjab (Ref No. D/165/FIMS), data was collected from different government and private universities of Lahore. Participants were explained about the nature and purpose of study. Participant's written informed consent was taken. They were assured that their information will be kept confidential and will be used only for research purpose. Demographic questionnaire and tools were then administered on the participants. A total of 328 participants were approached from which eight refused to participate in the study.

### 4.6 Statistical analysis

The data was analyzed using Statistical Package for Social Sciences (Version 25.0). Demographic data was analyzed using frequencies and percentages. Pearson product moment correlation was done to determine the association between demographic characteristics, subscales of multidimensional perfectionism scale, mattering scale and loneliness scale. Hierarchical multiple regression analysis was done to determine predictors for loneliness in young adults after controlling for covariates (age, closer relationship with father, mother and friends). Independent sample *t*-test was employed to find out gender differences on perfectionism, mattering and loneliness in young adults.

## 5 Results

The demographic characteristics of the participants showed that approximately 84 % male were enrolled in bachelors whereas 93 % females were enrolled in bachelors. Seventy three percent male participants were day scholars however, 84 % female participants were day scholars. Seventy one percent male participants lived in nuclear family system however, 81 % female participants lived in nuclear family system. Majority of participants had a satisfactory relationship with parents and friends (see Table 1).

**Table 1:** Frequencies and percentages of characteristics of the participants (N = 320).

Characteristics	Male (n = 160)		Female (n = 160)	
	*f*	%	*f*	%
Education				
Bachelors	135	84.4	150	93.8
Masters	25	15.6	10	6.3
Residence				
Day Scholar	118	73.8	135	84.4
Hostel	42	26.3	25	15.6
Satisfaction with Grades				
Yes	110	68.8	113	70.6
No	50	31.3	47	29.4
Religion				
Islam	156	97.5	170	97.1
Christianity	2	1.3	5	2.9
Other	2	1.3	0	0
Family System				
Joint	45	28.1	29	18.1
Nuclear	115	71.9	131	81.9
Closer relationship with Father				
Unsatisfactory	7	4.4	4	2.5
Somewhat unsatisfactory	7	4.4	5	3.1
Neutral	26	16.3	22	13.8
Somewhat satisfactory	23	14.4	13	8.1
Satisfactory	93	58.1	110	68.8
Late	4	2.5	6	3.8
Closer relationship with Mother				
Unsatisfactory	6	3.8	2	1.3
Somewhat unsatisfactory	5	3.1	4	2.5
Neutral	23	14.4	19	11.9
Somewhat satisfactory	13	8.1	16	10.0
Satisfactory	112	70.0	119	74.4
Late	1	0.6		
Home Atmosphere				
Unsatisfactory	9	5.6	4	2.5
Somewhat unsatisfactory	10	6.3	7	4.4
Neutral	23	14.4	23	14.4
Somewhat satisfactory	28	17.5	28	1\7.5
Satisfactory	90	56.3	98	61.3
Relationship with close friends				
Unsatisfactory	3	1.9	1	6
Somewhat	6	3.8	4	2.5
Unsatisfactory				
Neutral	17	10.6	7	4.4
Somewhat Satisfactory	25	15.6	18	11.3
Satisfactory	109	68.1	130	81.3
Attend parties and functions				
Never	3	1.9	3	1.9
Rarely	28	17.5	18	11.3
Sometimes	69	43.1	78	48.8
Often	60	37.5	61	38.1
Hang out with friends				
Never	1	0.6	3	1.9
Rarely	26	16.3	23	14.4
Sometimes	61	38.1	66	41.3
Often	72	45.0	68	42.5

A Kolmogorov-Smirnov test indicated that scores on multidimensional perfectionism scale, mattering and loneliness scales were normally distributed. Pearson product moment correlation was employed. Results showed that age (p < .01) and closer relationship with father (p < .01) were significantly negatively correlated with loneliness. The closer relationship with mother (p < .01) and friends (p < .05) were significantly negatively correlated with loneliness.

Self-oriented perfectionism, other-oriented perfectionism and socially prescribed perfectionism had significant positive correlation with loneliness (p < .01). Results also revealed that mattering (p < .01) had significant negative correlation with loneliness (see Table 2).

**Table 2:** Inter-correlations, mean, standard deviations for demographics, perfectionism, mattering and loneliness (N = 320).

	Variables	1	2	3	4	5	6	7	8	9	M	SD
1	Age	–	−.05	.07	.02	−.12*	−.18**	−.16**	.13*	−.21**	20.76	1.56
2	Relationship with father		–	.55**	.25**	−.13*	−.07	−.09	.04	−.09*	4.04	1.30
3	Relationship with mother			–	.30**	−.14**	−.07	−.09	.13**	−.09*	4.05	1.22
4	Relationship with friends				–	−.04	−.09	−.08	.09	−.10*	4.57	0.86
5	Self-Oriented Perfectionism					–	.86**	.86**	−.68**	.79**	74.55	15.98
6	Other-Oriented Perfectionism						–	.93**	−.74**	.87**	75.93	18.67
7	Socially Prescribed Perfectionism							–	−.72**	.88**	77.88	18.65
8	Mattering								–	−.73**	95.21	8.68
9	Loneliness									–	60.54	14.10

Note: **p < .01; *p < .05.

Hierarchical multiple regression (Enter method) was computed to determine predictors for loneliness in young adults after controlling for covariates. Age, relationship with father, relationship with mother and relationship with friends were entered as predictors of loneliness in Step 1, self-oriented perfectionism, socially prescribed perfectionism, other oriented perfectionism and mattering were entered as predictors of loneliness in Step 2. Step 1 predicted loneliness in young adults indicating 6 % of variance with age, F (4, 315) = 5.02, *p* < .001. This showed that young adults who were older in age were less likely to feel loneliness. Step 2 predicts loneliness in young adults indicating 75 % of variance with self-oriented perfectionism, socially prescribed perfectionism, other oriented perfectionism and mattering, F (8, 311) = 164.12, *p* < .000. The adults with more perfectionist tendencies were more likely to experience loneliness. Similarly, adults who perceived themselves to matter less, experienced more loneliness (see Table 3).

**Table 3:** Hierarchical multiple regression predicting loneliness from perfectionism and mattering.

	Loneliness	
Variables	*ΔR*^*2*^	*β*
Step 1	.06**	
Age		−.21***
Closer relationship with Father		−.08
Closer relationship with Mother		−.01
Closer relationship with Friends		−.07
Step 2	.75***	
Self oriented perfectionism		.32***
Other oriented perfectionism		.36***
Socially Prescribed perfectionism		.43***
Mattering		−.17***
*Total R*^*2*^	.81***	

*Note.* N = 320. *β* = Standardized Co-efficient.

*p < .05; **p < .01; ***p < .001.

Table 4 showed no significant gender differences on perfectionism, mattering and loneliness.

**Table 4:** Independent sample *t*-test showing gender differences on perfectionism, mattering and loneliness.

Variables	Men (n = 160)		Women (n = 160)				95 % CI		*Cohen's d*
	M	SD	M	SD	*t*(318)	p	LL	UL	
Self-oriented Perfectionism	74.53	15.32	73.58	16.67	−.03	.87	−3.57	3.47	0.06
Other Oriented Perfectionism	76.63	18.47	75.23	18.91	.67	.51	−2.72	5.51	0.07
Socially Prescribed Perfectionism	77.89	18.71	76.88	17.65	.01	.89	−4.09	4.12	0.05
Mattering	94.83	8.62	95.58	8.76	−.79	.43	−2.67	1.15	0.09
Loneliness	60.81	14.41	60.28	13.82	.34	.73	−2.57	3.64	0.04

*Note. M* = Mean; *SD* = Standard Deviation; *CI* =Confidence Interval; *LL* = Lower Limit; *UL* = Upper Limit.

## 6 Discussion

It was hypothesized that there is likely to be association of demographics variables, perfectionism, mattering and loneliness in young adults of Gen-Z. This hypothesis was partially accepted. Results revealed that age was significantly negatively correlated with loneliness which means that adults with increasing age are less likely to feel lonely. As, with increasing age come more responsibilities the young adults get occupied with their lives [2]. Young adulthood is a period of transition and the individual is supposed to take on new roles and responsibilities. They used to make new relationships which get strengthened and are sustained with the increasing age fulfilling the basic human need of intimacy and relatedness [21] which in turn diminish the feelings of loneliness in them.

Present study results also revealed that the young adults who had closer relationship with their friends are less likely to feel lonely. Research has found that the relationship with friends fulfills the basic need of young adults to form new relationships which diminished the feeling of loneliness in them [21]. Moreover, in Pakistani culture where collectivism prevails, relationships are given considerable importance. So, the attention young adults receive from friends fosters a sense that others are aware of their presence.

It was also hypothesized that perfectionism and mattering is likely to be predict loneliness in young adults after controlling for covariates. The hypothesis was fully accepted. Results revealed that mattering negatively correlated and predicted loneliness which means that the young adults who feel that they do not matter are more likely to experience loneliness. This can be related to a study who reported that low feelings of mattering to others was associated with loneliness in males and females [16]. Flett et al. reported that negative appraisal of mattering promotes social disconnection and isolation from others. As, the young adulthood is a phase of life concerned with forming and preserving relationships [12]. Mattering to others creates a sense of belongingness and connectedness to others [10] which is a basic need of young adults and when this need is not met, they tend to feel lonely [21].

Self-oriented perfectionism, socially prescribed perfectionism and other-oriented perfectionism positively correlated and predicted loneliness. This can be supported by studies who reported significant positive relationship between perfectionism and loneliness [9,22]. Perfectionist adults view themselves as impersonator who must present a perfect and flawless persona to the world. They strive hard to maintain their persona due to which even their accomplishments seems unfulfilling to them and they feel lonely [23]. Moreover, adults with elevated standards for themselves often expect others to be as critical as themselves which impedes their ability to communicate successfully with others and form close friendships which engender loneliness in them. Literature also suggests that perfectionism and loneliness often co-occur [24]. This may be due to the fact that individuals high on perfectionistic tendencies get too busy in achieving high standards in their goals accomplishment that they are unable to maintain or do not want to form interpersonal relationships which causes them to feel loneliness eventually. Previous literature also supports the significant positive relationship between perfectionism and loneliness [9,22]. 10.13039/100014337Furthermore, in Pakistan where culture is collectivistic, people prefer to interact and socialize with others. So, they strive to get approval of others which then encourages them for an ideal self-presentation [25]. So, they set elevated standards for themselves and strive for flawlessness which leads them to experience loneliness.

It was also hypothesized that there is likely to be gender difference on perfectionism, mattering and loneliness in young adults. The hypothesis was not accepted. No gender differences were found in the perfectionism [26] and loneliness [27] which is consistent with the previous literature who reported no significant differences were found between men and women on mattering [9,28]. This may be due to the fact that in Pakistani culture the roles for men and women were earlier well defined and most women did not have the privilege to go to university and participate in social life. But, today with increasing knowledge and advancement, the well-defined roles for men and women are dissolving. Men and women today are facing same pressures and challenges. The university-oriented lifestyle is becoming popular and women, like men, are also seeking better career opportunities.^29^ Moreover, the pressure associated with the impositions of adulthood regarding independence, education, employment, and making new relationships [2] are also same for both genders so it could also be the reason contributing to no significant differences in the scores of perfectionism, mattering, and loneliness in them. Concisely, due to same challenges and demands of the modern era both men and women set similar elevated standards and when these standards are not met they tend to experience loneliness.

## 7 Conclusion

It can be concluded that perfectionist individuals of Gen-Z tend to set stringent standards for themselves and others as well. The more they engage in perfectionism, the more they tend to feel that they do not matter to others. Feeling of not mattering to others can possibly lead the person to experience loneliness.

## 8 Limitations

The study has several limitations. First of all, the study targeted the population of young adults (18–24 years) so, results are not generalizable across various age ranges. Future studies can be conducted so that the phenomena can be studied across wider age ranges. Moreover, the present study was a cross sectional study, it allowed us to study the relationship of perfectionism, mattering and loneliness but it doesn't allow us to infer causal relationship. So, a longitudinal study can be conducted to further explore the phenomenon. The present study included the individuals whose relationship status was single. So, in future a comparison study can be conducted between single and married individuals.

## 9 Future and practical implications

This study will provide insight regarding the role of mattering along with perfectionism and loneliness in the Pakistani culture where interpersonal relationships and social engagement for young adults are emphasized. The study will also provide the understanding to the perfectionists of how their need to be perfect can possibly lead them to experience loneliness.

There is a likelihood that a person who feels lonely might be at risk of developing problems like suicidal ideation and even attempts so the present study can be used at preventive level as well.

## Data availability statement

Data associated with this study was not submitted to publicly available repository. Data will be made available on request.

## Funding

Not funded

## CRediT authorship contribution statement

**Bushra Shafiq:** Writing - review & editing, Writing - original draft, Methodology, Formal analysis, Data curation, Conceptualization. **Anam Ali:** Writing - review & editing, Writing - original draft, Methodology, Formal analysis, Conceptualization. **Hidna Iqbal:** Writing - review & editing, Writing - original draft, Methodology, Conceptualization.

## Declaration of competing interest

The authors declare that they have no known competing financial interests or personal relationships that could have appeared to influence the work reported in this paper.
